# Supplementary material for: Mapping the breast cancer metastatic cascade onto ctDNA using genetic and epigenetic clonal tracking
Source: Nat Commun. 2020 Mar 27;11:1446. doi: 10.1038/s41467-020-15047-9 (PMC7101390; doi:10.1038/s41467-020-15047-9)
Supplement: Supplementary file 2 — Supplementary Information [file 41467_2020_15047_MOESM2_ESM.pdf]

Supplementary information

Mapping the breast cancer metastatic cascade onto ctDNA using genetic and epigenetic clonal tracking

George D Cresswell[1], Daniel Nichol[1], Inmaculada Spiteri[1], Haider Tari[1,2], Luis Zapata[1], Timon Heide[1], Carlo Maley[3], Luca Magnani[4], Gaia Schiavon[5], Alan Ashworth[6], Peter Barry[7], Andrea Sottoriva[1]

1. Evolutionary Genomics and Modelling Lab, Centre for Evolution and Cancer, The Institute of Cancer Research, London, UK.
2. Glioma Lab, The Institute of Cancer Research, London, UK.
3. Arizona State University, Tempe, AZ, USA.
4. Imperial College London, London, UK.
5. Oncology R&D, AstraZeneca, Cambridge, UK.
6. UCSF Helen Diller Family Comprehensive Cancer Center, 1450 3rd St, San Francisco, CA 94158, USA.
7. Department of Surgery, Breast Unit, Royal Marsden Hospital, London, UK.

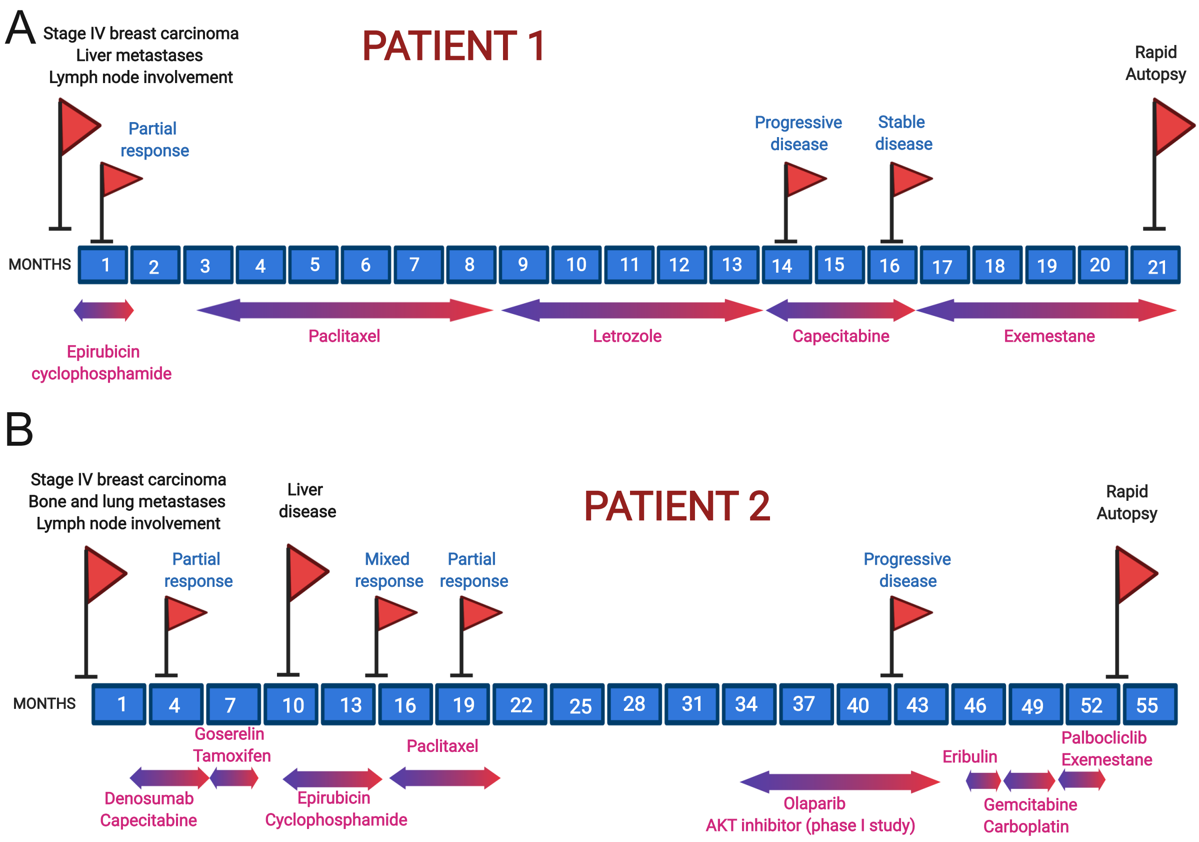

Supplementary Figure 1. Clinical history of LEGACY patients.

Chronological clinical history of Patient 1 (A) and Patient 2 (B).

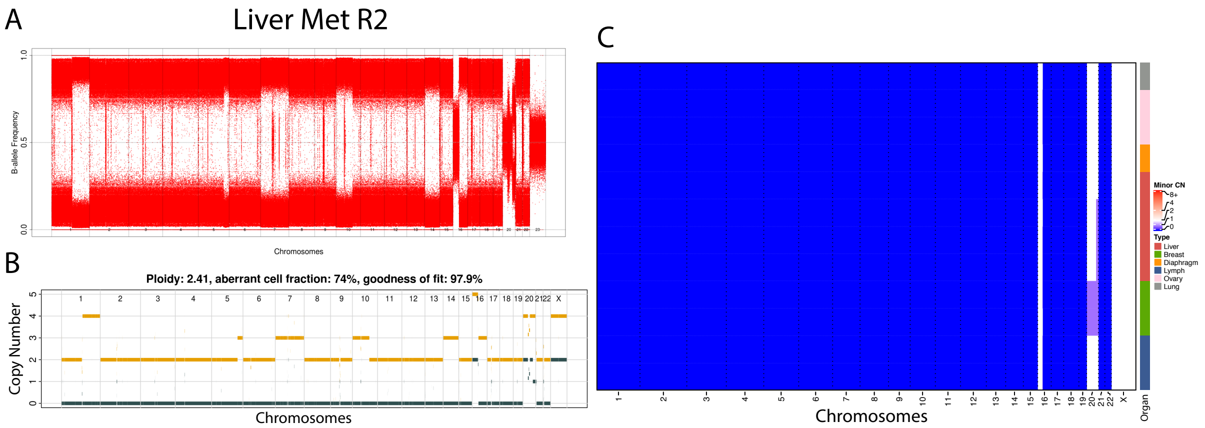

Supplementary Figure 2. Genome-Wide LOH in Patient 1.

(A) B-Allele frequency across the genome of LEGACY patient 1 indicates genome-wide loss of heterozygosity. (B) Allele-specific copy number analysis confirms a largely diploid genome, but with the major allele contributing both copies. (C) Minor allele heatmap shows that there are zero copies of the minor allele throughout the genome of this patient.

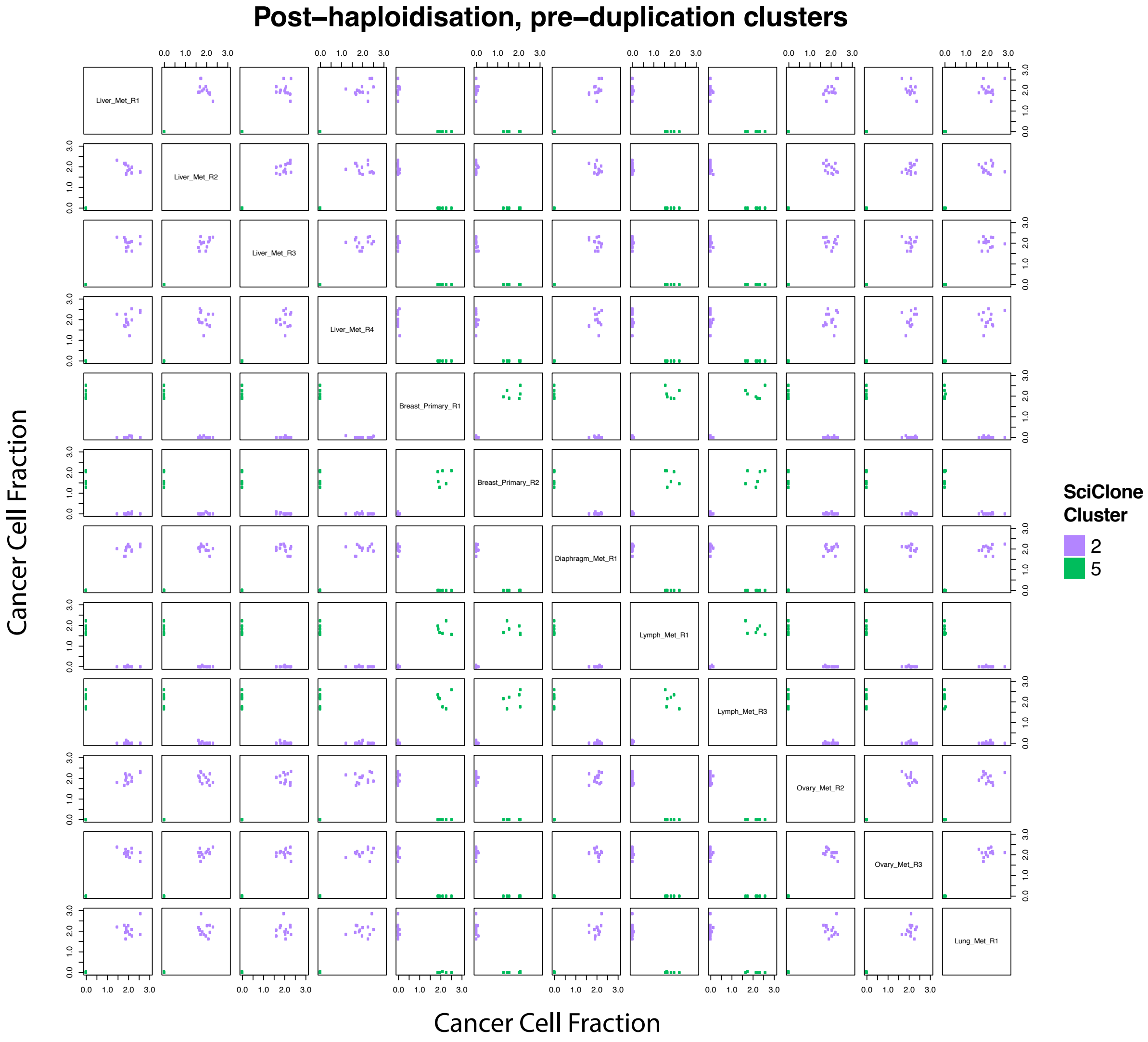

Supplementary Figure 3. Separate whole genome duplication clusters in Patient 1.

There were two separate clusters of SNVs that occurred before whole-genome reduplication but were not truncal, rather they were present in all primary and lymph node samples and in all metastases separately. The CCF-CCF distributions show the two clusters are entirely distinct.

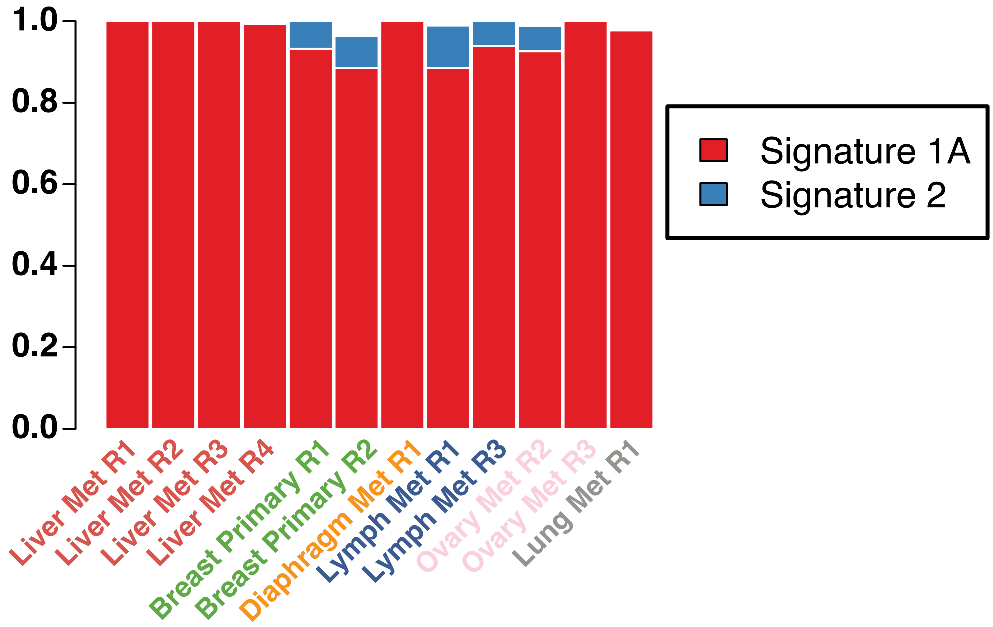

Supplementary Figure 4. Mutational signature analysis for Patient 1.

The dominant mutational process in this patient is cytosine deamination, which is the result of aging.

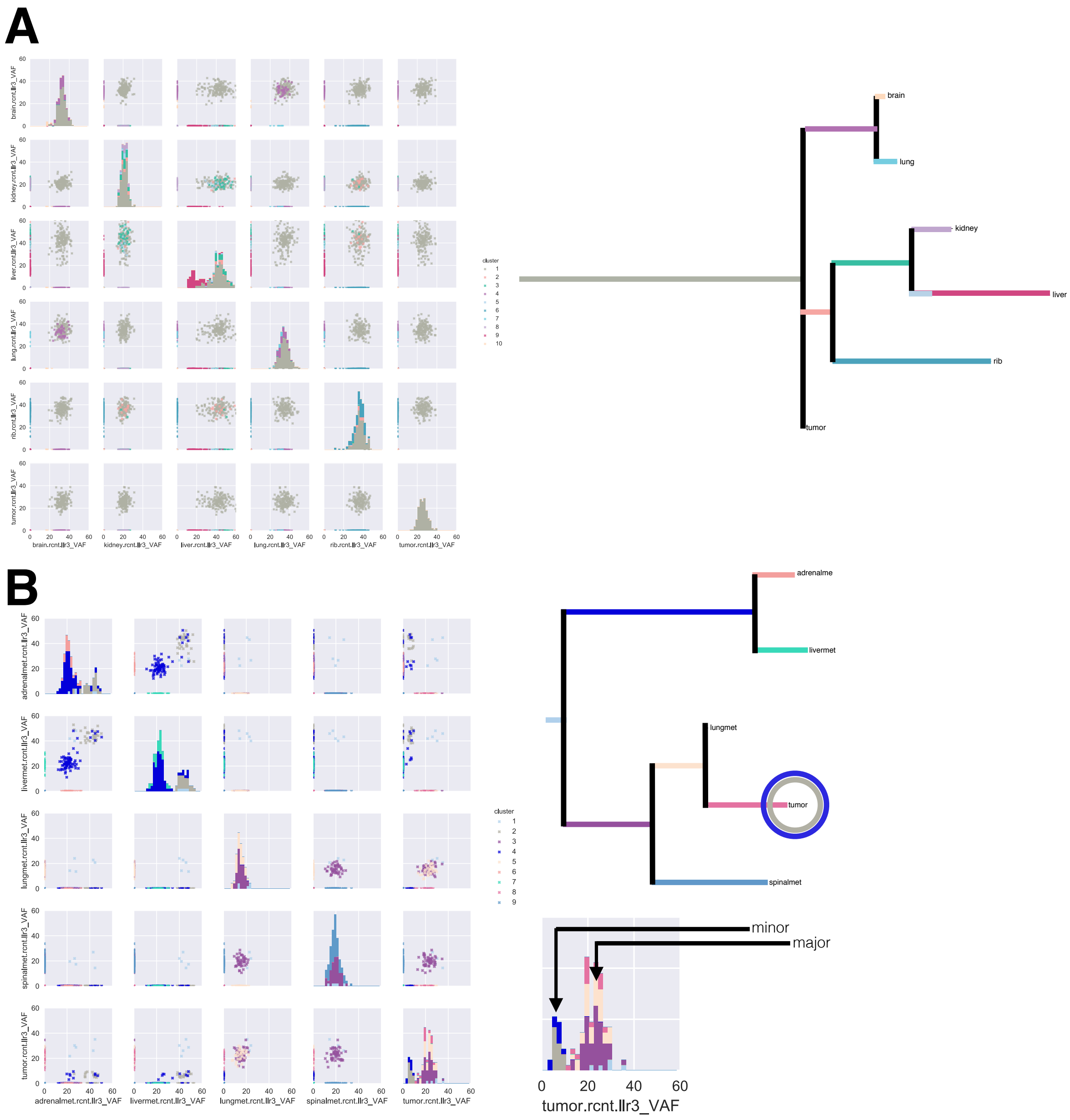

Supplementary Figure 5. Reanalysis of Hoadley et al.

We reanalysed Hoadley et al. 2016 using a stricter and more conservative bioinformatics analysis and confirmed that in Patient A7 (A) and Patient A1 (B) all lesions were of monoclonal origin. Previously inferred polyclonality was supported by few mutant reads of unclear significance and confounded by missed copy number events.

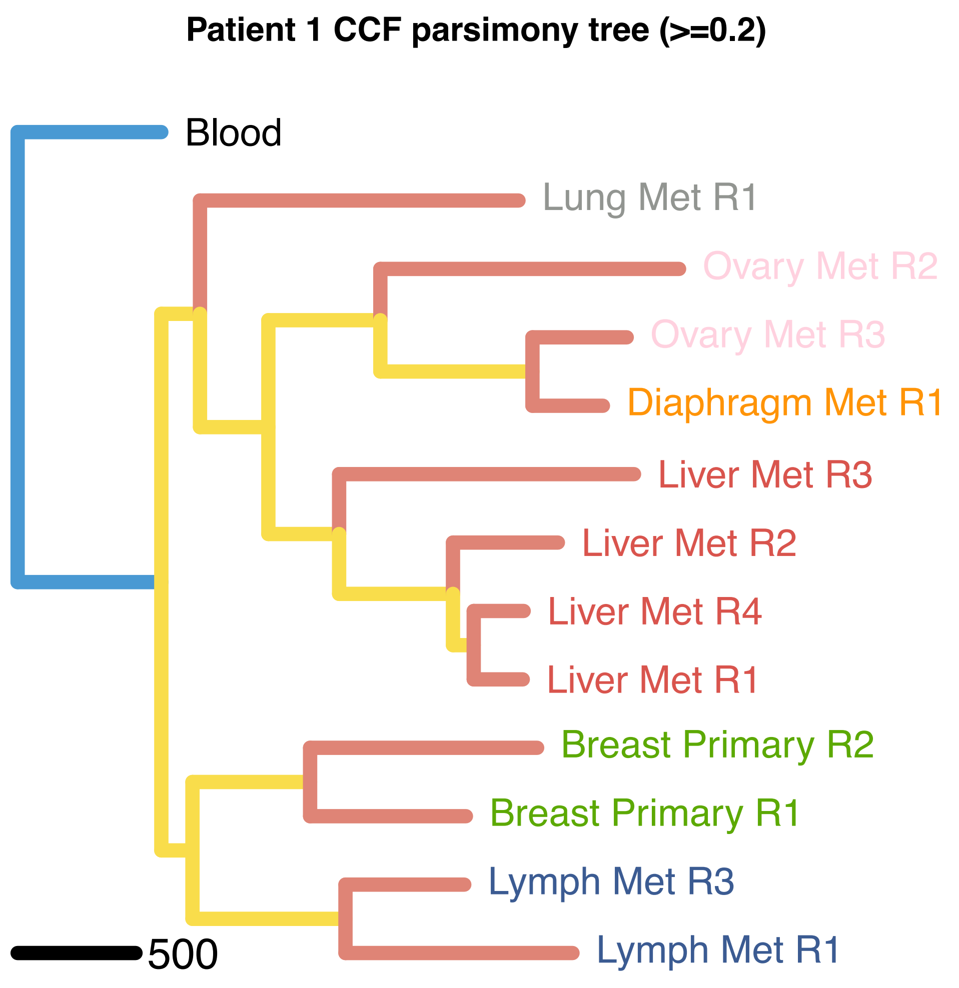

Supplementary Figure 6. Sample tree for patient 1.

We reconstructed the phylogenetic tree for this patient using parsimony prior to subclonal deconvolution.

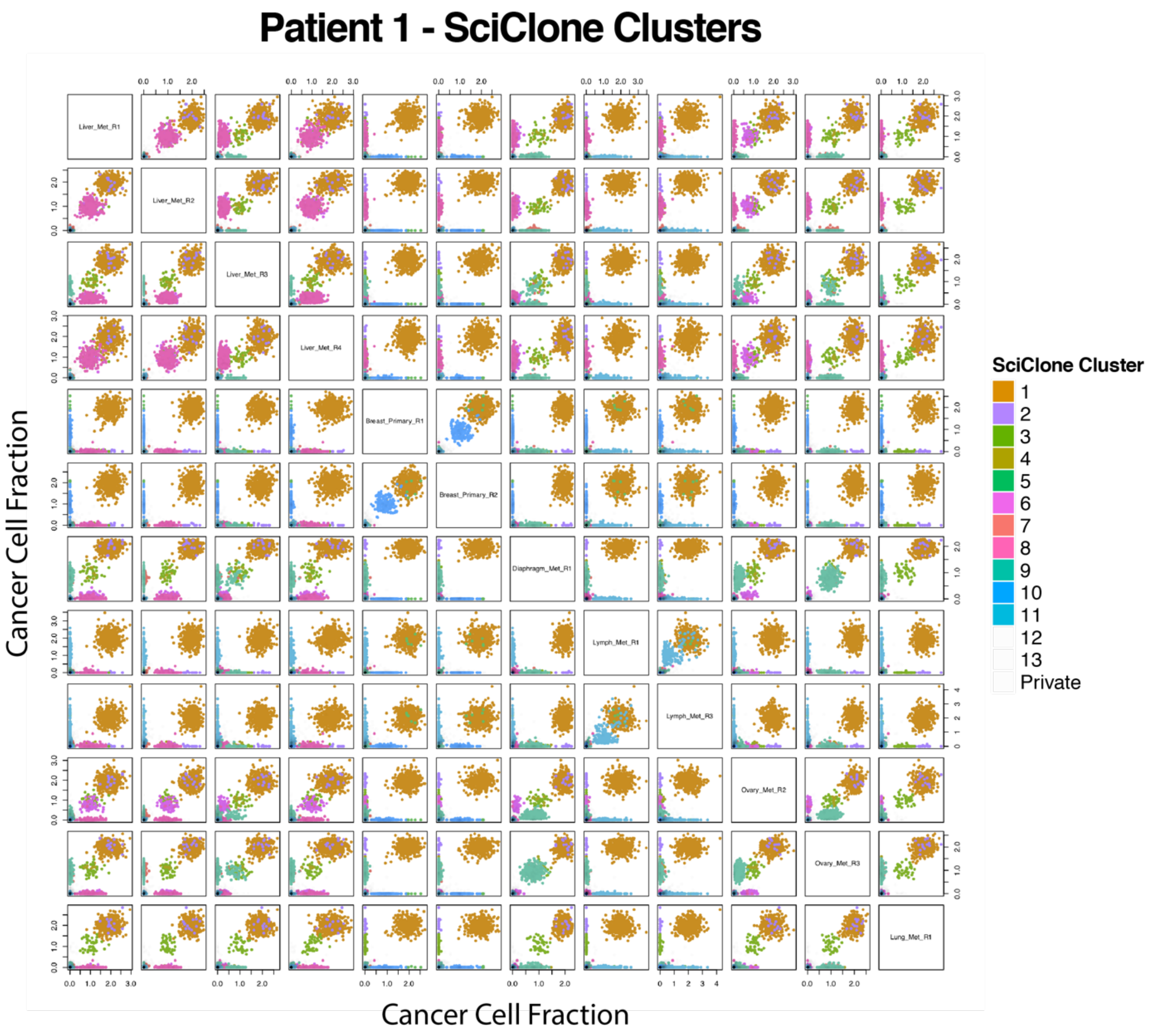

Supplementary Figure 7. 2D Cancer Cell Fraction plots of Patient 1.

All mutational clusters plotted for all samples from LEGACY patient 1 following sciClone analysis.

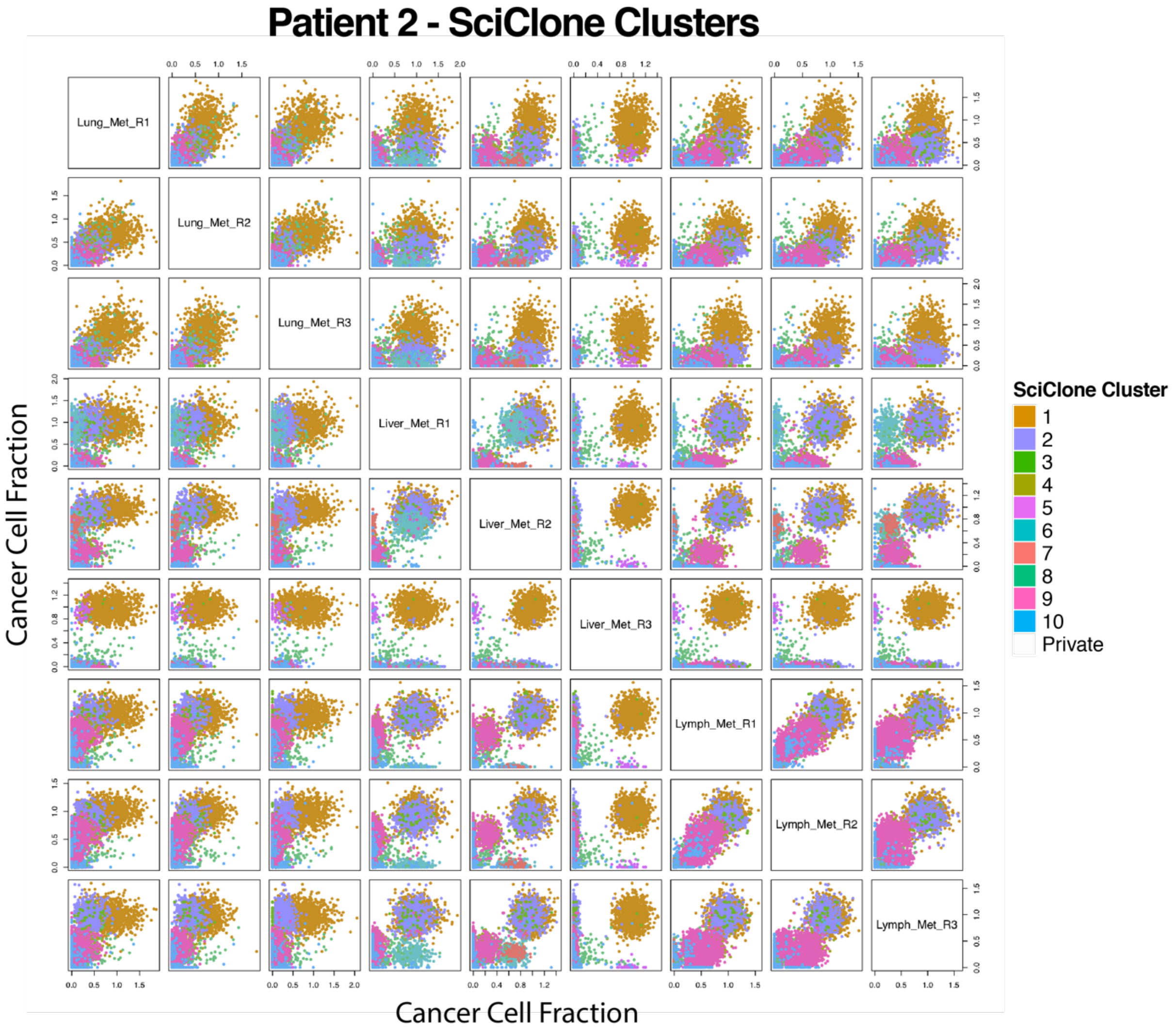

Supplementary Figure 8. 2D Cancer Cell Fraction plots of Patient 2.

All mutational clusters plotted for all samples from LEGACY patient 2 following sciClone analysis.

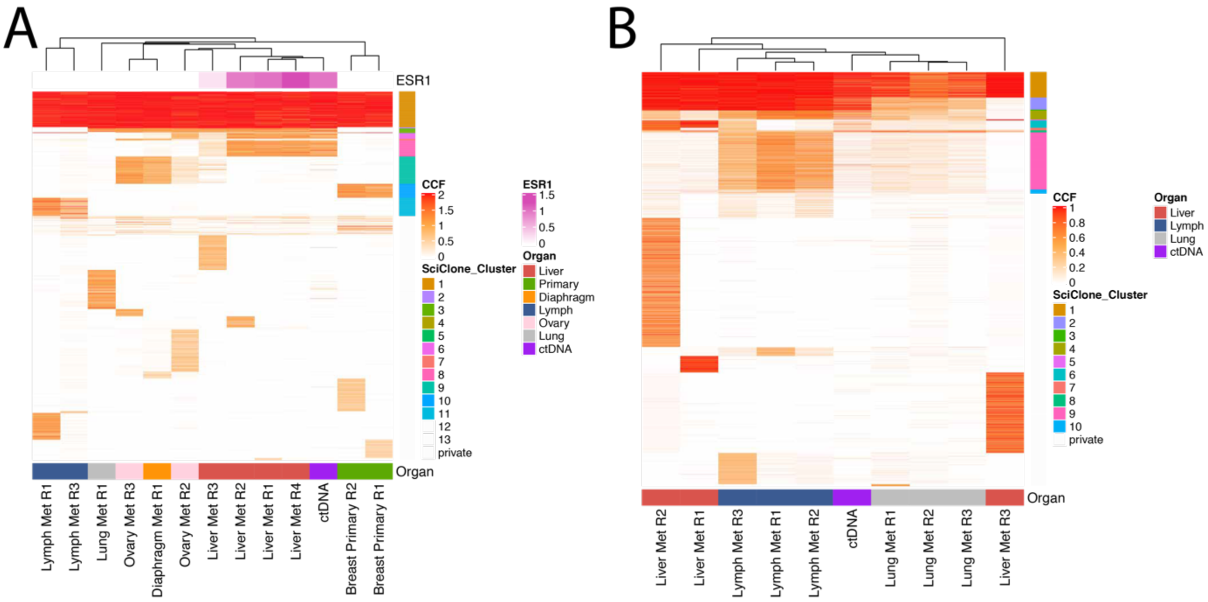

Supplementary Figure 9. CCF heatmaps including ctDNA.

Heatmap of CCF clusters that includes ctDNA as a sample for Patient 1 (A) and Patient 2 (B). Patterns reflect the analysis in Figure 4.

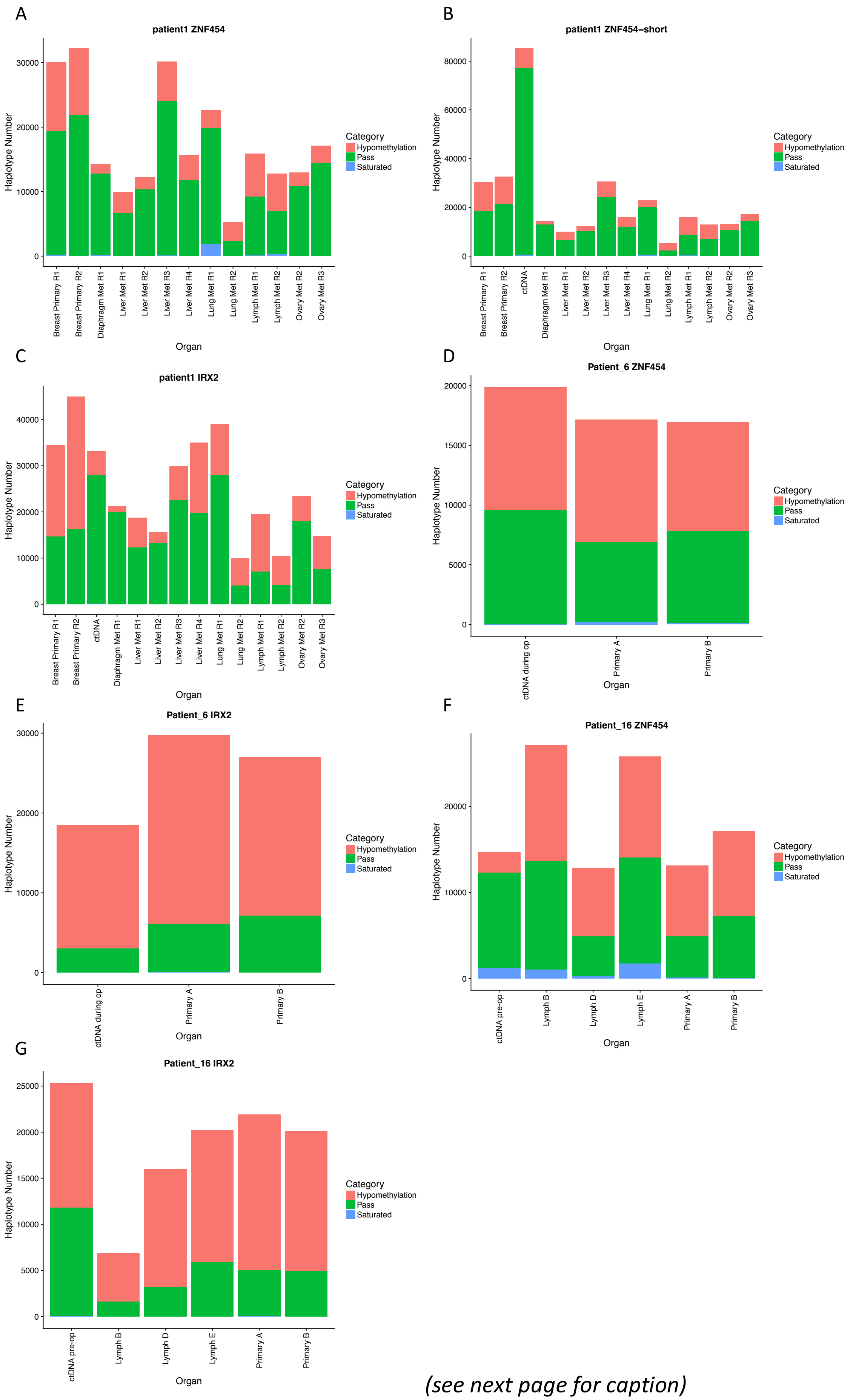

(see next page for caption)

Supplementary Figure 10. Quality control of the methylation haplotypes.

Hypomethylated (in our case completely unmethylated) haplotypes are probably deriving from non-cancer cells and were excluded from our analysis. Saturated (>80% methylated) haplotypes were also excluded. Here we show the results for ZNF454 (A), the short version of ZNF454 (B) and IRX2 (C) for Patient 1 and ZNF454 and IRX2 for Patient 6 (D and E respectively) and Patient 16 (F and G respectively) from Barry et al. 2018.

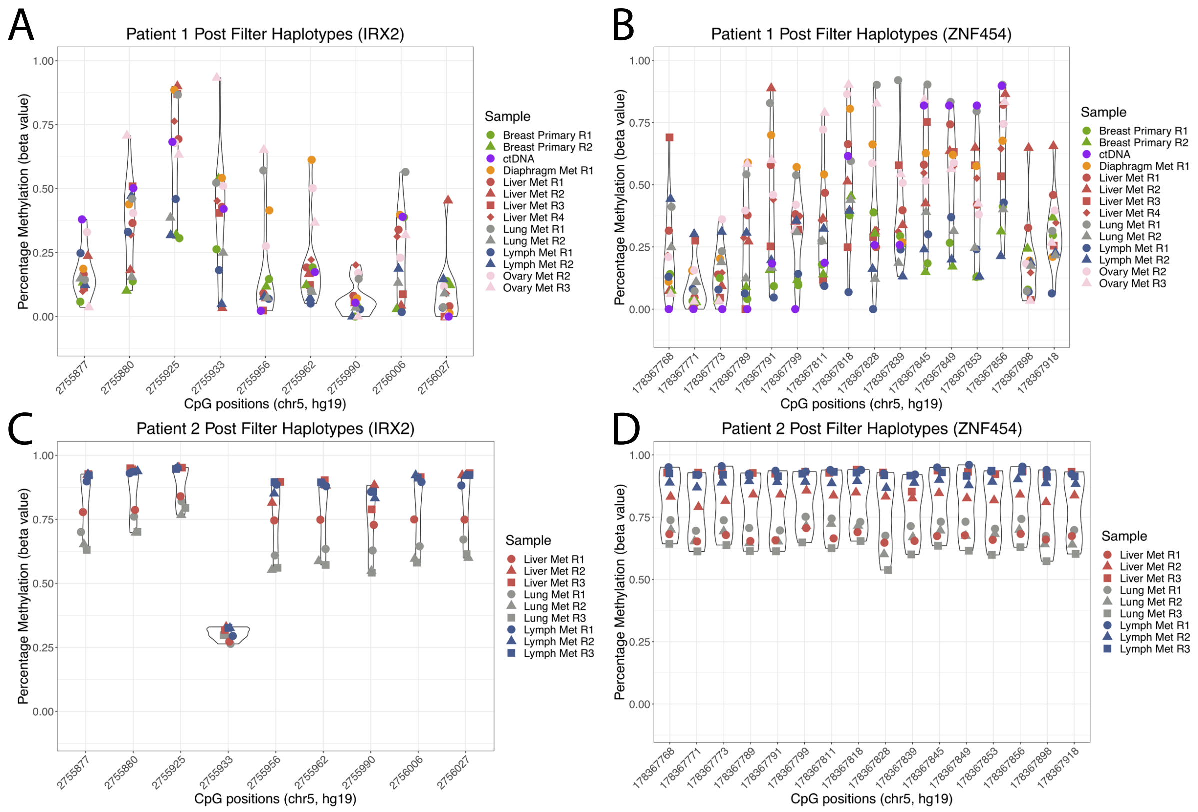

Supplementary Figure 11. Percent methylation for each CpG per sample.

For each CpG in each clock we plot the average methylation in each sample. Whereas for Patient 1 the data showed a good signal of haplotype variability in both IRX2 (A) and ZNF454 (B), for Patient 2 the patterns were characterised by hypermethylation in almost all CpGs in both IRX2 (C) and ZNF454 (D). Patient 1 was filtered for hypo- and hypermethylation. To represent hypermethylation, Patient 2 is only filtered for hypo-methylation.

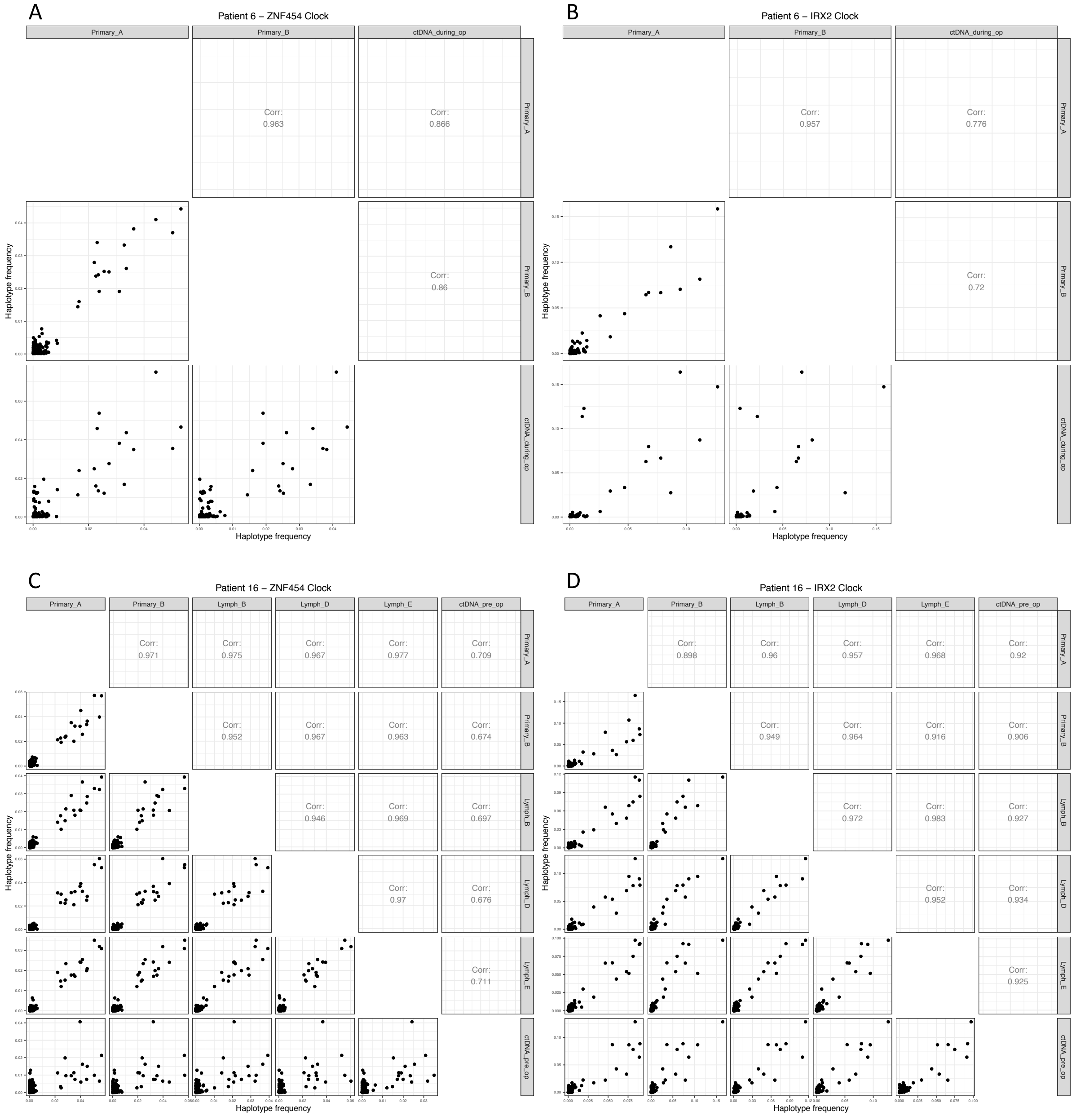

Supplementary Figure 12. Correlation between haplotype frequencies from an orthogonal cohort.

We generated new methylation clock data from an orthogonal cohort of early breast cancers with matched tissue and ctDNA previously presented (Barry et al. 2018). Having only a couple of tissue samples and matched ctDNA we performed a simpler analysis of correlation between haplotype frequencies in the ctDNA and in each of the tissue samples. Plots show significant correlations between solid biopsies and ctDNA in this cohort, confirming the robustness of clocks in a separate independent cohort. Panels display clocks ZNF454 and IRX2 for Patient 6 (A and B, respectively) and Patient 16 (C and D, respectively).
