# Supplementary material for: Mapping the breast cancer metastatic cascade onto ctDNA using genetic and epigenetic clonal tracking
Source: Nat Commun. 2020 Mar 27;11:1446. doi: 10.1038/s41467-020-15047-9 (PMC7101390; doi:10.1038/s41467-020-15047-9)
Supplement: Supplementary file 3 — Description of Additional Supplementary Files [file 41467_2020_15047_MOESM3_ESM.pdf]

Description of Additional Supplementary Files

File Name: Supplementary Data 1
Description: Copy number calls.

File Name: Supplementary Data 2
Description: SNV calls.

File Name: Supplementary Data 3
Description: Digital Droplet PCR results for PIK3CA variant in patient 1. Supplementary Data 4. SNV clustering assignments.

File Name: Supplementary Data 4
Description: SNV clustering assignments.

File Name: Supplementary Data 5
Description: Purity and ploidy estimates per sample.

File Name: Supplementary Data 6
Description: Predicted neoantigens of LEGACY patient 1. Supplementary Data 7. Single-molecule methylation haplotypes.

File Name: Supplementary Data 7
Description: Single-molecule methylation haplotypes.
